# A Novel *In Vitro* Method to Assess the Microbial Barrier Function of Tissue Adhesives Using Bioluminescence Imaging Technique

**DOI:** 10.1155/2022/3483238

**Published:** 2022-01-10

**Authors:** Yalda Mirzaei, Kerstin Hagemeister, Martina Hüffel, Timo Schwandt, René H. Tolba, Julia Steitz

**Affiliations:** ^1^Institute for Laboratory Animal Science, Faculty of Medicine, RWTH Aachen University, Aachen, Germany; ^2^Adhesys Medical GmbH, Aachen, Germany; ^3^Institutes of Molecular Medicine and Experimental Immunology (IMMEI), University of Bonn, Bonn, Germany

## Abstract

*Background*. Tissue glues can minimize treatment invasiveness, mitigate the risk of infection, and reduce surgery time; ergo, they have been developed and used in surgical procedures as wound closure devices beside sutures, staples, and metallic grafts. Regardless of their structure or function, tissue glues should show an acceptable microbial barrier function before being used in humans. This study proposes a novel *in vitro* method using *Escherichia coli Lux* and bioluminescence imaging technique to assess the microbial barrier function of tissue glues. Different volumes and concentrations of *E. coli Lux* were applied to precured or cured polyurethane-based tissue glue placed on agar plates. Plates were cultured for 1 h, 24 h, 48 h, and 72 h with bioluminescence signal measurement subsequently. Herein, protocol established a volume of 5 *μ*L of a 1 : 100 dilution of *E. coli Lux* containing around 2 × 10^7^ CFU/mL as optimal for testing polyurethane-based tissue glue. Measurement of OD_600nm_, determination of CFU/mL, and correlation with the bioluminescence measurement in p/s unit resulted in a good correlation between CFU/mL and p/s and demonstrated good reproducibility of our method. In addition, this *in vitro* method could show that the tested polyurethane-based tissue glue can provide a reasonable barrier against the microbial penetration and act as a bacterial barrier for up to 48 h with no penetration and up to 72 h with a low level of penetration through the material. Overall, we have established a novel, sensitive, and reproducible *in vitro* method using the bioluminescence imaging technique for testing the microbial barrier function of new tissue glues.

## 1. Introduction

For decades, sutures, metallic ware, and staples have been used as the main method to achieve wound closure or internal implant fixation in surgical setups [1, 2]. However, these methods can cause a deficiency of tissue integration, significant mismatch between tissue and fixation, leakage, and additional trauma leading to surgical site infection (SSI) by microorganisms [3, 4]. Tissue glue as a physical barrier device against microorganism penetration can provide a fast and noninvasive option with easier application method compare to the traditional invasive techniques [5]. Tissue glues can be categorized mainly as fibrin-, cyanoacrylate-, or polyurethane-based glues regarding to their structure or as hemostats, sealants, and adhesives based on their intended use and application [6, 7]. A tissue glue as an adhesive should have strong wet adhesion properties and be stable under different physiological conditions. Tissue adhesives, if being used as topical wound closure devices, benefit from less risk of needle stick injury [8]. As a result, this will decrease the rate of suture tract infections and fluid or air leakage [9, 10]. Polyurethane-based tissue adhesives are a family of surgical glues that are entirely manufactured of a synthetic polyurethane prepolymer and an amine-based curing agent. Both components are provided in a single-use, two-chambered ready-to-use syringe. As soon as the prepolymer mixes with the curing agent, the poly-addition reaction begins. Surgical site infection caused by microbial contamination of the wound site is the most occurring healthcare-associated infection [11]. Immediately upon occurring a defect in the stratum corneum barrier of the epidermis, organisms commonly responsible for wound infections adhere to the surface of the injured tissue and initiate an infection [12]. The subsequent development of this infection is related to both the size of the initial bacterial inoculum and the success of local and systemic defense mechanisms of the living being [13, 14]. In consideration of that, a tissue adhesive as a wound closure device should have an acceptable microbial barrier function. To prove the microbial barrier effectiveness of tissue adhesives and demonstrate their microbial barrier function, an *in vitro* assessment based on the Bhende's methodology has been conducted and described before [15, 16]. The basis of the test is subjecting the adhesive layer in contact with various organisms responsible for surgical site infections [17]. In this manner, a wound closure device that forms a barrier for up to 72 hours provides sufficient time for the natural wound healing process and evaluated to be effective [15, 18]. Recently, the advancement of genetic engineering methods allows scientists introducing bioluminescence genes from numerous species into the bacteria, cells, and animals that can further be used in *in vitro* and *in vivo* studies by applying new imaging techniques [19]. In the work described herein, we have utilized transduced *Escherichia coli* with a *Lux* gene that enables emitting bioluminescent light without the necessity of adding a luciferin substrate [20, 21]. By doing so, the bacterial growth and subsequently the microbial barrier function can be evaluated through bioluminescence imaging, a nontoxic and highly sensitive analytical technique that has been used in a wide range of studies on living cells and animals [22, 23]. In the presented study, we established a novel *in vitro* method to assess the microbial barrier function of a polyurethane-based tissue adhesive.

## 2. Materials and Methods

The study was divided into three parts to address: (i) the adherence ability of a polyurethane-based tissue adhesive to contact surfaces like agar or petri dishes and subsequently the growth of bacteria as pretest 1, (ii) the optimal dosage and bioluminescence signal of the bacteria culture as pretest 2, and (iii) the microbial barrier function of a polyurethane-based tissue adhesive as main study.

### 2.1. *E. coli Lux* Culture

*Escherichia coli Lux* was generated and kindly provided by Dr. Timo Schwandt. Culture was prepared using bacteria glycerol stock added in to 3 mL sterile Standard I Nutrient Broth medium (Carl Roth GmbH + Co. KG, Karlsruhe, Germany) containing 100 *μ*g/mL Ampicillin. The culture was incubated overnight at 37°C in an incubator shaker with 190 rpm.

### 2.2. Optical Density Determination

The growth of overnight *E. coli Lux* culture was checked the next day by visual control of the culture cloudiness, then was determined by the optical density measurement using a spectrophotometer at the wavelength of 600 nm (*BioPhotometer®*, *Eppendorf*, *Hamburg*, *Germany*). OD_600_ of around three was considered as optimal.

### 2.3. Titration Assay and Bioluminescence Measurement

A serial dilution of 1 : 2 was performed by applying 200 *μ*L of the overnight *E. coli Lux* culture collected at OD6_00_ around three to a 96-well microplate. Subsequently, bioluminescence measurement was employed using the in vivo imaging system with the exposure time between 0.5 second and 1 min (*IVIS® Lumina XR II*, *Caliper Life Sciences*, *Inc*., *Hopkinton*, *MA*, *USA*).

### 2.4. Colony Forming Unit (CFU) Determination

To determine the colony forming unit (CFU)/mL, 50 *μ*L of serial dilutions (10^4^, 10^5^, and 10^6^) of the overnight *E. coli Lux* culture was cultured on Tryptone Blood Sheep Soy Agar (TBSA) plates (*Oxoid ™ Deutschland GmbH*, *Wesel*, *Germany*). Plates containing 30–300 colonies were counted to calculate the final bacterial concentration as CFU/mL values.

### 2.5. The Polyurethane-Based Tissue Adhesive Preparation

In pretest 1, the polyurethane-based tissue adhesive (PU-glue) was cured as a single layer on the inner side of the sterile aluminum pouch according to the instruction for clinical application. The adhesive strips had approximately 2 mm thickness and were remained under the laminar flow hood for 2-3 minutes allowing them to polymerize. 2 × 2 cm squares of the PU-glue were prepared using a sterile scalpel. Additionally, 2 × 2 cm squares of sterile filter paper were used as control. The cured PU-glue as well as sterile filter papers was placed on agar plates and on plain petri dishes. Furthermore, the PU-glue was used as its liquid form (uncured) and applied directly to a petri dish. In pretest 2 and the main study, the polyurethane-based tissue adhesive was solely used in its cured form and placed directly on TBSA plates.

### 2.6. Inoculation

In pretest 1, a total of 10 cured and 10 uncured PU-glue in addition to control groups for each set were tested. 5 of each group were inoculated with 5 *μ*L and 5 others with 10 *μ*L of pure *E. coli Lux* culture. In pretest 2, 5 *μ*L of different dilutions of the *E. coli Lux* culture (1 : 10, 1 : 100, and 1 : 1000) was used for inoculation. TBSA plates with PU-glue or control filter paper were incubated at 37°C for 1, 24, 48, and 72 hours. At each time point, they were evaluated for morphology (visual control of bacterial growth and shape of the glue/filter) and subsequently the bioluminescence signal measurement according to the imaging protocol.

### 2.7. Imaging Protocol

Bioluminescence imaging was performed using an IVIS® Lumina XR II imaging system (*Caliper Life Sciences*, *Inc*., *Hopkinton*, *MA*, *USA*). The plates were placed in the specimen chamber, and photon emission was measured with the following settings: Binning: 8, Aperture (f/stop): 1, field of view (FOV): D, and the subject height: one centimeter. The bioluminescence values were measured without using the excitation/emission filter (open) and at room temperature. The exposure time was set ranging from 0.5 second to 1 min depending on the bioluminescence intensity signal. Afterwards, the region of interest (ROI) tool was used to determine the total number of photons detected per second (photons/s) on the specific parts of the test specimens. For each test and control plate for each time interval, three different images were obtained: (i) from above, which measured the whole surface of the PU-glue/filter papers; (ii) after removal of the PU-glue/filter papers, which measured only the area below of the glue or filter paper, not the bacteria growth around them; and (iii) cross-section which measured the half-cut surface of PU-glue/filter papers placed perpendicularly on the TBSA plate.

### 2.8. Data Analysis

Data analyses were performed using the Living Image® 4.7.3, Microsoft Excel and GraphPad Prism 8 (GraphPad Software Inc., San Diego, CA, USA) software package for windows 10. The total flux signal in photons per second from a specific site (site of testing article or filter paper) was quantified by defining a region of interest (ROI) drawn manually, then measured using the Living Image® Software. Henceforward, the background levels were obtained by measuring the total flux signal from a ROI (outside the test material or filter paper). Background values were then subtracted from the measurement values followed by calculation of the mean value and standard deviation of each replicate. Moreover, the radiance signal was measured in photons/second/cm^2^/steradian (p/sec/cm^2^/sr). Statistical calculations and quality assessment of different rounds of experiments were performed using the GraphPad Prism software. The data obtained from all measurements were converted to CFU/mL values according to the linear regression analysis and the correlation graph. From there, two-way ANOVA with Bonferroni posttest was performed, and the effects were considered statistically significant if *p* ≤ 0.05.

## 3. Results

### 3.1. Pretest 1

**Figure 1:**
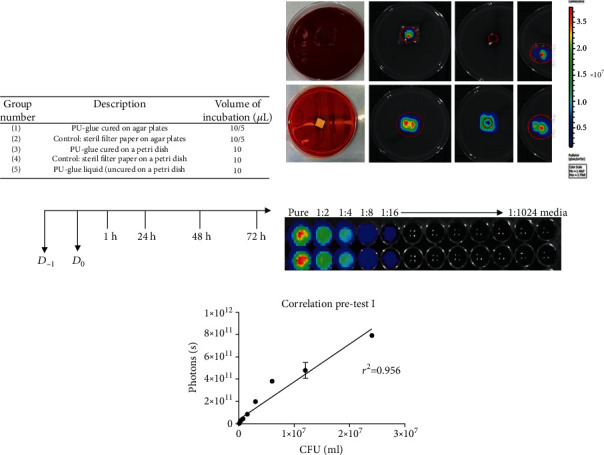
Experiment design of pretest 1: (a) group assignment; (b) representative pictures of the bioluminescence imaging from above, bottom site, and cross section indicating the light intensity with the red color as the most intense and the blue color as the least intense signal and the set region of interest (ROI). (c) Experimental design D_−1_: start of the *E. coli Lux* culture overnight 1 day before of experiment; D_0_: testing of *E. coli Lux* culture by optical density (OD_600nm_), titration assay, and CFU/mL measurement and application of *E. coli Lux* to test and control articles. 1 h, 24 h, 48 h, 72 h: assessment of morphology (bacterial growth), adhesion of PU-glue/sterile filter papers, and bioluminescence imaging. (d) Image of *E. coli Lux* titration (1 : 2 dilution series, row A/B-1 to A/B-10). Values in A/B-12 represent measurements of the control (media only). (e) Linear regression graphs for *E. coli Lux* titration and bioluminescence results and calculated *r*^2^ values.

**Figure 2:**
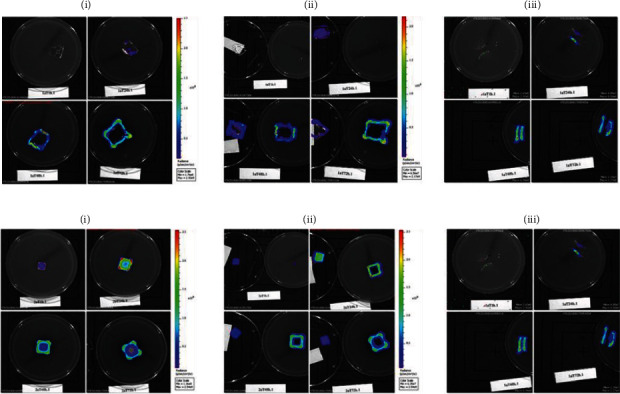
Results of bioluminescence imaging after inoculation with 10 *μ*L *E. coli Lux*: (a) culture on PU-glue and (b) culture on control filter paper after 1 h, 24 h, 48 h, and 72 h time points from (i) above, (ii) bottom, and (iii) cross section, respectively. Bioluminescence signal is shown as radiance (p/sec/cm^2^/sr) on a color-coded intensity scale depicted in the color bars beside the image. For the cross section images ((iii) in (a, b)), Min and Max values of the measurement range are indicated on the bottom right corner of the images.

**Figure 3:**
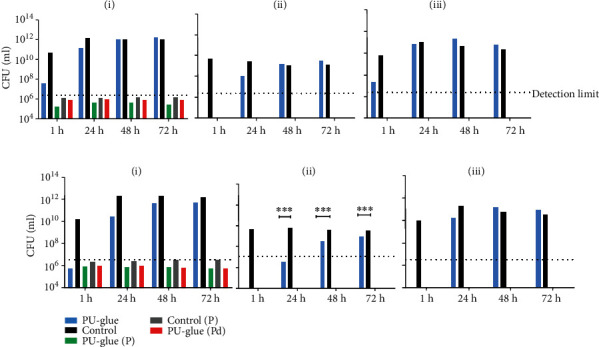
Results of bioluminescence measurement converted to CFU/mL: (a) after inoculation with 10 *μ*L *E. coli Lux* culture. (b) After inoculation with 5 *μ*L *E. coli Lux* culture on test materials at the indicated time points and sites: (i) above, (ii) bottom, and (iii) cross section. PU-glue: cured and placed on agar plates; Control: sterile filter paper placed on agar plates; PU-glue (P): cured and placed on a petri dish; Control (P): sterile filter paper placed on a petri dish, PU-glue (Pd) liquid (uncured) on a petri dish. Dotted line indicates the detection limit. ^∗∗∗^*p* < 0.0005, *n* = 1.

The adherence ability of polyurethane-based adhesive to the contact surfaces of TBSA plate and petri dish and subsequently the bacterial growth was assessed in pretest 1. Furthermore, two different volumes of *E. coli Lux* culture (5 and 10 *μ*L) were tested by visual control and bioluminescence signal measurement (group assignment, see Figure 1(a), *n* = 1). Signals were measured at three different sites (above, bottom, cross-section) as shown in Figure 1(b) at different time points as depicted in the experimental design in Figure 1(c). For verification of the *Lux* gene expression, the bioluminescence signals from different dilutions of *E. coli Lux* were first measured in a titration assay (Figure 1(d)). Afterwards, these results were correlated with the colony forming units' values evaluated using standard culture methods. High correlation in the indicated range with a correlation coefficient of *r*^2^ = 0.956 was demonstrated (Figure 1(e)). Representative pictures of the bioluminescence measurement with 10 *μ*L of incubated *E. coli Lux* culture on TBSA plates after 1 h, 24 h, 48 h, and 72 h at the indicated sites are shown in Figure 2. In general, there was no visible bacterial growth and no measurable bioluminescence signal in groups 3, 4, and 5 on the condition that the test materials were just placed on petri dishes. In group 1, after 1 h of incubation, no bioluminescence signal could be measured, whereas a small signal was detected in the control group. However, as the incubation time reached to 72 h, the bioluminescence signals increased in both groups (1 and 2). After 48 h, a spillover effect/overgrowth of bacteria was detected with a high intensity at the edges resulting from a faster bacteria growth when directly in contact with the agar. To prevent this effect, only 5 *μ*L inoculation of *E. coli Lux* culture was tested in pretest 2 and the main study. Results of bioluminescence measurements converted to colony forming unit values after inoculation with 10 and 5 *μ*L of *E. coli Lux* culture are shown in Figure 3. Here, the calculated CFU/mL values of PU-glue placed on agar plates showed detectable levels starting from 1 h of incubation reaching to the highest level at 24 h with 10 *μ*L of *E. coli Lux* (Figure 3(a)). No significant differences between PU-glue and control groups could be seen at different time points measured from the above, the bottom side, and cross sections. However, the CFU/mL values of PU-glue group after 48 h and 72 h were higher than that of the control group in cross section measurements. Using 5 *μ*L of *E. coli Lux* culture resulted in more prominent discrimination between control and PU-glue as shown in Figure 3(b). Bioluminescence signals converted into CFU/ml from PU-glue placed on agar plates showed detectable levels starting from 24 h of incubation, whereas in control group the detectable bioluminescence signal started after 1 h and reached to the highest level at 24 h. Significant differences between PU-glue and control groups could be seen on the bottom site measurements until 72 h of inoculation. No bioluminescence signals above the detection limit could be measured in the groups in which PU-glue or filter papers were placed on petri dishes.

### 3.2. Pretest 2

**Figure 4:**
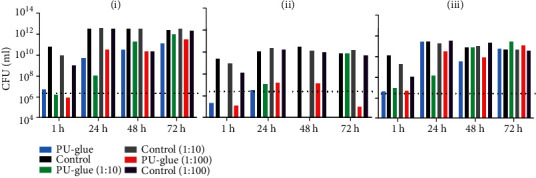
Results of bioluminescence measurement converted to CFU/mL. After inoculation of 5 *μ*L *E. coli Lux* culture with different dilutions on test materials at the indicated time points and sites: from (i) above, (ii) bottom, and (iii) cross section. PU-glue and control: inoculation with pure *E. coli Lux*. PU-glue (1 : 10) and control (1 : 10): inoculation with 1 : 10 dilution of *E. coli Lux*. PU-glue (1 : 100) and control (1 : 100): inoculation with 1 : 100 dilution of *E. coli Lux*. There was no detectable bacterial growth with 1 : 1000 dilution of *E. coli Lux*.

To further optimize our method, 5 *μ*L aliquots of three different dilutions (1 : 10, 1 : 100, and 1 : 1000) from overnight *E. coli Lux* culture were tested on PU-glue and control filter papers in pretest 2. Bioluminescence signals were measured and converted into colony forming units at the indicated dilutions and are shown in Figure 4. In the PU-glue group, after 1 h incubation with different dilutions of *E. coli Lux*, no visible bacteria growth and no measurable bioluminescence signals were detectable. In the control group, only minor bacteria growth could be observed at this time point. Hereinafter, bioluminescence signals of PU-glue group with pure, 1 : 10, and 1 : 100 dilution of *E. coli Lux* showed a detectable level starting after 24 h of incubation. The signal reached to the highest level as the incubation time increased to 72 h. On the contrary, in the control group with pure, 1 : 10, and 1 : 100 dilution of *E. coli Lux*, the bioluminescence signal started to be detectable after 1 h of incubation and reached to its highest level at the 24 h time points. The 1 : 1000 dilution did not show any bacteria growth or measurable bioluminescence signal neither on the PU-glue nor on control filter paper. In general, differences between PU-glue and control groups could be only seen in measurement from above and on the bottom side of the test material at 1 h, 24 h, and 48 h time point. However, the differences were not significant due to the small sample size. Clear differences between PU-glue and control groups could be detected at 1 h and 24 h time points from cross section measurement. Nonetheless, these differences decreased afterwards at 48 h and 72 h.

### 3.3. The Main Study

In the main study, the polyurethane-based adhesive was investigated further for its microbial barrier function according to the method developed and optimized in pretests 1 and 2 while using cured adhesive film and 5 *μ*L of a 1 : 100 dilution of *E. coli Lux* on agar plates were established and considered as the optimal approach. For standardization and comparability of our method, the measured CFU/mL values, OD_600nm_, photons per second (p/s) values, and calculated correlation coefficients (between CFU/mL and p/s values) for pretest 1, pretest 2, and the main study are shown in Table 1. The compared data indicate that bacteria cultures with an optical density (OD_600nm_) around 3, which will result in comparably similar CFU/mL values and show a good correlation with the measured bioluminescence signals (p/s).

**Figure 5:**
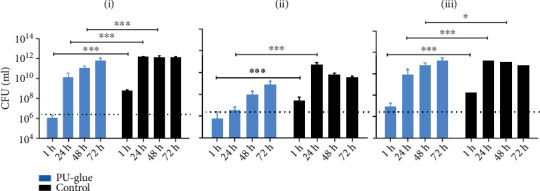
Results of bioluminescence measurement converted to CFU/mL. After inoculation of 5 *μ*L *E. coli Lux* culture with 1 : 100 dilution in test materials at the indicated time points and sites: (i) above, (ii) bottom, and (iii) cross section. ^∗∗∗∗^*p* < 0.0001, ^∗^*p* < 0.05, *n* = 10.

**Figure 6:**
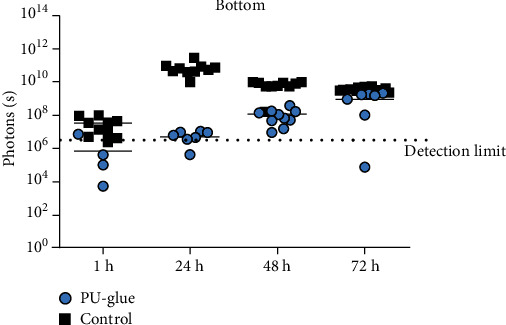
Scattered dot plot of the bioluminescence measurement results in photon/s. After inoculation of 5 *μ*L *E. coli Lux* culture with 1 : 100 dilution in test materials at the indicated time points and sites: (i) above, (ii) bottom, and (iii) cross section.

For the evaluation of the PU-glue in the main study, we compared 10 test articles vs. control using the above established method (Figure 5). All 10 test articles retained their integrity as a microbial barrier up to 72 h when measured by visual observation. All controls were positive as evident by visual observation and bioluminescence measurements at 1 h, 24 h, 48 h, and 72 h. In general, bacterial growth according to the bioluminescence measurement from above was significantly reduced after 24 h in the PU-glue group, indicating a slower bacterial growth. Measuring the bioluminescence intensity from the bottom side, significant differences between PU-glue and control groups were observed at 1 h and 24 h of incubation. However, the difference was not significant at 48 h and 72 h since the signal levels in the control group decreased. Furthermore, the cross-section data showed significant differences between PU-glue and control groups at 24 h, 48 h, and 72 h time points, similar to the measured data from above. In total, the data from this *in vitro* model indicate the penetration of *E. coli Lux* through the tested PU-glue after 48 h. It could be also demonstrated that the polyurethane-based tissue adhesive is an effective barrier to microbial penetration with 95% confidence only for 24 h based on the obtained data in the main study. The scattered dot plot data illustrated in Figure 6 show details about the single measurement from each plate. Interestingly, bacterial growth after 24 h of incubation was negatively measured from some plates in PU-glue group, whereas after 48 h all plates became positive using the bioluminescence measurement. This underlines the need of at least 10 test articles per group at each time point.

## 4. Discussion

The discomfort and pain caused by invasive techniques such as sutures, staples, and metallic ware and the potential accompanied surgical site infection raise the need for developing new methods that can minimize treatment invasiveness, mitigate the infection, and reduce surgery time [24–26]. Since decades, tissue adhesives have been addressed as an attractive alternative for wound closure or internal implant fixation in surgical setups, providing a microbial barrier and even antimicrobial properties [27]. Wound healing is a complex process and requires several orderly events to occur simultaneously. A natural barrier to outside elements will be formed within 48 hours through epithelial cell generation and basal cell movement across the incised dermis [28]. Until this time, the injured tissue has little or no tensile strength and is solely dependent on the wound closure device to provide a suitable barrier and maintain its integrity [29]. Thereby, new wound closure devices such as tissue adhesives should be tested for their microbial barrier function, antimicrobial, and removal properties. The purpose of here-described study was to establish a novel *in vitro* method to assess the microbial barrier function of tissue adhesives using the bioluminescence imaging technique. We employed *E. coli Lux* bacteria, the bioluminescent form of *Escherichia coli*, as one of the organisms commonly responsible for wound infection to evaluate the microbial barrier function of a polyurethane-based tissue adhesive [19, 20, 30, 31]. This family of adhesives is known to be fully synthetic, nontoxic, flexible, and biodegradable with a certain barrier strength [7, 24, 32]. In pretest 1, the adherence ability of PU-glue to the contact surfaces of agar or petri dish, its shape, and appearance were assessed by utilizing two different volumes of the *E. coli Lux* culture. Data showed that unlike the clinical situation, the PU-glue will not remain plane especially on agar plates during the incubation period. As a consequence, this characteristic can interfere with the final results. Additionally, the agar media plate is needed to observe bacterial growth. Therefore, the best application method for *in vitro* assessment of PU-glue was curing the PU-glue as a layer on the inner side of the sterile aluminum pouch and placing it on the agar plate after polymerization for further assessment. In pretest 2, 5 *μ*L of different bacteria dilutions was used and analyzed. Data indicated that the 1 : 100 dilution (corresponding to at least 2 × 10^7^ CFU/mL) is sensitive enough to detect differences between the test and the control group and show a reasonable bacterial growth over time. The data from pretests 1 and 2 confirmed the importance of finding the right volume and concentration of the bacteria culture and knowing about the behavior of the test material to establish a reliable and reproducible method to discriminate efficiently between test materials. To avoid the observed spillover effect and overgrowing of bacteria due to large volume and amount, the 5 *μ*L volume of 1 : 100 dilution (10^7^ CFU/mL) was selected as the optimum. As seen in Table 1, the measured CFU/ml, OD_600nm_, p/s, and calculated correlation coefficients (between CFU and p/s measurement) showed a good standardization of our method when OD_600nm_ level of 3 is reached in the bacterial culture. Based on our experience, it is necessary to have a good bacteria stock management and to work with the one prior selected clone that showed the highest intensity in bioluminescence measurement, since the bioluminescence signals can vary among bacteria clones. Regarding the bioluminescence analyses, data are presented as pseudo-color images showing the light intensity superimposed over the grayscale reference. The red color in these images is indicating the most intense region, and the blue color defines the least intense one. However, it has to be noticed that no optical signal in the region of interest does not necessarily mean no bioluminescence result, especially if stronger signals are detected elsewhere in the picture. Thus, a separate ROI analysis is needed. Furthermore, the bioluminescence detection limit has to be evaluated in bacteria culture titration assay using background level measurements of the test materials. All in all, working with the same bacterial stock, proper storing, selecting the best clone, and antibiotic treatment are crucial criteria to reach a standard and reproducible method. Furthermore, the photon per second values from imaging measurements needs to be converted to CFU/mL to have rational results and normalized data for evaluation. However, the data from cross section images are not suitable for assessing the microbial barrier function of the here used polyurethane-based tissue adhesive due to its biodegradable characteristics over time which results in penetration of the bacteria. The resolution and sensitivity of the measurement are not high enough to discriminate between the bacterial growth on the material and the bacterial growth through it. On this account, measuring the bacterial growth below the tissue adhesive as an indication of its ability to prevent the bacteria from reaching to the skin surface is considered as an assessment of microbial barrier function. Results of the main study indicated the penetration of *E. coli Lux* through the PU-glue after 48 h of incubation. Comparing the here described *in vitro* method with standard *in vivo* models, it should be noted that this novel *in vitro* assessment is much more sensitive than an animal infection model, since in practice higher numbers of organisms would be required to generate an infection in an *in vivo* model owing to the inherent immune response [17]. Moreover, the imaging settings play an important role in obtaining standard and accurate results which are reproducible. The amount of the collected light, the distance from the lens to the sample, subject height, and the exposure time are some of the factors which can affect the results. One limitation of this *in vitro* method would be that it does not simulate the wound healing process; thus, it cannot be studied through this model. Additionally, the agar plate may not reflect the clinical situation since it is different from the skin [33].

However, the results of this study can easily be extended to other bacteria species responsible for SSIs (*Staphylococcus aureus*, *Pseudomonas aeruginosa*, *Staphylococcus epidermidis*, and *Enterococcus faecalis*) [34]. Since many years, the role and significance of microorganisms in wound healing are discussed. On the one hand, it is thought that microbial density is of primary importance in wound infection [18, 35–38]; on the other hand, it has been argued that the types of microorganisms to be of greater importance [39–44]. Despite the fact that wound environment is mostly poly-microbial [45–50], one study indicates one-third of the total number of microbial species in colonized wounds are anaerobic. Moreover, this frequency and prevalence increases to approximately 50% if the wound is infected [51]. Several studies also determined that the majority of infected wounds are colonized with at least 10^5^ CFU/g of bacteria [36, 52–58]. It has always been a matter of concern to strictly compare *in vitro* results from evaluating studies on tissue adhesives since the bacterial strains used, the inoculated volume, and CFU/mL values were different [33]. Bioluminescence bacteria have been utilized in many *in vivo* studies and showed many potentials to be used in different fields of science [59–63]. The goal of the here-described study was to observe, detect, and measure actively growing bacteria using bioluminescence imaging techniques in an *in vitro* situation as a novel method to assess the microbial barrier function. The established *in vitro* assessment not only provides easier and earlier detection of bacterial growth but also more reliable data to evaluate the microbial barrier function of a tested tissue adhesive.

## 5. Conclusion

The purpose of this study was establishing a novel *in vitro* method to evaluate the microbiological barrier function of tissue adhesives using bioluminescent imaging technique. Data of the *in vitro* experiment supported the hypothesis that the tested polyurethane-based tissue adhesive can provide a reasonable barrier to microbial penetration with 95% confidence for 24 h. The results suggest that this PU-glue is likely to act as a bacterial barrier for up to 48 h with no penetration and up to 72 h with a low level of penetration, proven by the low levels of bacterial growth.

## Figures and Tables

**Table 1:** Characterization of the E. coli Lux bacteria culture. Optical density measurement (OD_600nm_), the measured and calculated CFU/mL values, the mean of bioluminescence signals measured in 5 *μ*L of a 1 : 2 *E. coli Lux* dilution, and correlation coefficient between colony forming units and photon per second values as *r*^2^ are shown.

Test round	OD_600 nm_	CFU/mL	Mean (p/s)	*r* ^2^ (CFU/mL vs. p/s)
Pretest 1	3.011	2.4 × 10^7^	7.76*E*+10	0.956
Pretest 2	3.065	2.48 × 10^7^	1.29*E*+09	0.8919
Main study	3	2.66 × 10^7^	1.46*E*+10	0.9510

## Data Availability

The datasets during and/or analyzed during the current study are available from the corresponding author on reasonable request.

## References

[1] Weiser T. G., Haynes A. B., Molina G. (2015). Estimate of the global volume of surgery in 2012: an assessment supporting improved health outcomes. *The Lancet*.

[2] Weiser T. G., Regenbogen S. E., Thompson K. D. (2008). An estimation of the global volume of surgery: a modelling strategy based on available data. *The Lancet*.

[3] Zhang W., Ji T., Lyon S. (2020). Functionalized multiarmed polycaprolactones as biocompatible tissue adhesives. *ACS Applied Materials & Interfaces*.

[4] Lauto A., Mawad D., Foster L. J. R. (2008). Adhesive biomaterials for tissue reconstruction. *Journal of Chemical Technology & Biotechnology*.

[5] Dompé M., Vahdati M., van Ligten F. (2020). Enhancement of the adhesive properties by optimizing the water content in PNIPAM-functionalized complex coacervates. *ACS Applied Polymer Materials*.

[6] Spotnitz W. D. (2014). Fibrin sealant: the only approved hemostat, sealant, and adhesive—a laboratory and clinical perspective. *International Scholarly Research Notices*.

[7] Reece T. B., Maxey T. S., Kron I. L. (2001). A prospectus on tissue adhesives. *The American Journal of Surgery*.

[8] Dumville J. C., Coulthard P., Worthington H. V. (2014). Tissue adhesives for closure of surgical incisions. *Cochrane Database of Systematic Reviews*.

[9] Liebelt E. L. (1997). Current concepts in laceration repair. *Current Opinion in Pediatrics*.

[10] Quinn J., Drzewiecki A., Li M. M. (1993). A randomized, controlled trial comparing a tissue adhesive with suturing in the repair of pediatric facial lacerations. *Annals of Emergency Medicine*.

[11] Magill S. S., Hellinger W., Cohen J. (2012). Prevalence of healthcare-associated infections in acute care hospitals in Jacksonville, Florida. *Infection Control and Hospital Epidemiology*.

[12] Arbuthnott J. P., Smyth C. J., Ellwood D. C., Melling J., Rutter P. (1979). Bacterial adhesion in host-pathogen interactions in animals. *Adhesion of Microorganisms to Surfaces*.

[13] Edberg S. C., Neter E. (1981). Methods of quantitative microbiological analyses that support the diagnosis, treatment, and prognosis of human infection. *CRC Critical Reviews in Microbiology*.

[14] Krizek T. J., Robson M. C. (1975). Evolution of quantitative bacteriology in wound management. *The American Journal of Surgery*.

[15] (2016). Effectiveness of cyanoacrylate topical skin adhesives as a microbial barrier protectant. https://www.cardinalhealth.com/content/dam/corp/web/documents/whitepaper/CardinalHealth-Topical-Skin-Adhesives-Antimicrobial-Whitepaper.pdf.

[16] Brown L. (2014). *Effectiveness of Indermil® Tissue Adhesive as a Microbial Barrier*.

[17] Bhende S., Rothenburger S., Spangler D. J., Dito M. (2002). In vitro assessment of microbial barrier properties of Dermabond® topical skin adhesive. *Surgical Infections*.

[18] Mangram A. J., Horan T. C., Pearson M. L., Silver L. C., Jarvis W. R. (1999). Guideline for prevention of surgical site infection, 1999. *American Journal of Infection Control*.

[19] Meighen E. A. (1991). Molecular biology of bacterial bioluminescence. *Microbiology and Molecular Biology Reviews*.

[20] Welsh D. K., Noguchi T. (2012). Cellular bioluminescence imaging. *Cold Spring Harbor Protocols*.

[21] Hutchens M., Luker G. D. (2007). Applications of bioluminescence imaging to the study of infectious diseases. *Cellular Microbiology*.

[22] Gregor C., Gwosch K. C., Sahl S. J., Hell S. W. (2018). Strongly enhanced bacterial bioluminescence with theiluxoperon for single-cell imaging. *Proceedings of the National Academy of Sciences*.

[23] Welsh D. K., Kay S. A. (2005). Bioluminescence imaging in living organisms. *Current Opinion in Biotechnology*.

[24] Duarte A., Coelho J. F., Bordado J. C., Cidade M. T., Gil M. H. (2012). Surgical adhesives: systematic review of the main types and development forecast. *Progress in Polymer Science*.

[25] Ninan L., Monahan J., Stroshine R. L., Wilker J. J., Shi R. (2003). Adhesive strength of marine mussel extracts on porcine skin. *Biomaterials*.

[26] Liu Y., Meng H., Konst S., Sarmiento R., Rajachar R., Lee B. P. (2014). Injectable dopamine-modified poly (ethylene glycol) nanocomposite hydrogel with enhanced adhesive property and bioactivity. *ACS Applied Materials & Interfaces*.

[27] Rushbrook J. L., White G., Kidger L., Marsh P., Taggart T. F. (2014). The antibacterial effect of 2-octyl cyanoacrylate (Dermabond®) skin adhesive. *Journal of Infection Prevention*.

[28] Hunt T. K., Englebert Dunphy J. (1979). Fundamentals of wound management. *Normal Repair*.

[29] Quinn J. V. (1998). *Tissue adhesives in wound care*.

[30] Brodl E., Niederhauser J., Macheroux P. (2018). *In Situ* measurement and correlation of cell density and light emission of bioluminescent bacteria. *Journal of Visualized Experiments*.

[31] (2021). *Bacterial Transformation Is The Process Of Transferring Bacteria*.

[32] Ferreira P., Pereira R., Coelho J. F. J., Silva A. F. M., Gil M. H. (2007). Modification of the biopolymer castor oil with free isocyanate groups to be applied as bioadhesive. *International Journal of Biological Macromolecules*.

[33] Stoffel J., Bernatchez S. F. (2017). Effect on microbial growth of a new skin protectant formulation. *Advances in Wound Care*.

[34] Giacometti A., Cirioni O., Schimizzi A. M. (2000). Epidemiology and microbiology of surgical wound infections.. *Journal of Clinical Microbiology*.

[35] Heggers J. (1998). Defining infection in chronic wounds: does it matter?. *Journal of Wound Care*.

[36] Heggers J. P., Robson M. C., Doran E. T. (1969). Quantitative assessment of bacterial contamination of open wounds by a slide technique. *Transactions of the Royal Society of Tropical Medicine and Hygiene*.

[37] Raahave D., Friis-Møller A., Bjerre-Jepsen K., Thiis-Knudsen J., Rasmussen L. B. (1986). The infective dose of aerobic and anaerobic bacteria in postoperative wound sepsis. *Archives of Surgery*.

[38] Robson M. C. (1999). Lessons gleaned from the sport of wound watching. *Wound Repair and Regeneration*.

[39] Danielsen L., Balslev E., Döring G. (1998). Ulcer bed infection. *APMIS*.

[40] Lavery L. A., Harkless L. B., Felder-Johnson K., Mundine S. (1994). Bacterial pathogens in infected puncture wounds in adults with diabetes. *The Journal of Foot and Ankle Surgery: Official Publication of the American College of Foot and Ankle Surgeons*.

[41] Madsen S. M., Westh H., Danielsen L., Rosdahl V. T. (1996). Bacterial colonization and healing of venous leg ulcers. *APMIS*.

[42] Pallua N., Fuchs P. C., Hafemann B., Völpel U., Noah M., Lütticken R. (1999). A new technique for quantitative bacterial assessment on burn wounds by modified dermabrasion. *Journal of Hospital Infection*.

[43] Schraibman I. G. (1990). The significance of beta-haemolytic streptococci in chronic leg ulcers. *Annals of the Royal College of Surgeons of England*.

[44] Sehgal S., Arunkumar B. (1992). Microbial flora and its significance in pathology of sickle cell disease leg ulcers. *Infection*.

[45] Brook I. (1989). Aerobic and anaerobic microbiology of Bartholin’s abscess. *Surgery, Gynecology & Obstetrics*.

[46] Brook I. (1989). Microbiology of postthoractomy sternal wound infection. *Journal of Clinical Microbiology*.

[47] Brook I. (1995). Microbiology of gastrostomy site wound infections in children. *Journal of Medical Microbiology*.

[48] Rosa R. D., Rosa E. D., Panichi G. (1996). Anaerobic bacteria in postsurgical infections: isolation rate and antimicrobial susceptibility. *Journal of Chemotherapy*.

[49] Nichols R. L., Smith J. W. (1994). Anaerobes from a surgical perspective. *Clinical Infectious Diseases*.

[50] Sanderson P., Wren M., Baldwin A. (1979). Anaerobic organisms in postoperative wounds. *Journal of Clinical Pathology*.

[51] Bowler P., Duerden B., Armstrong D. G. (2001). Wound microbiology and associated approaches to wound management. *Clinical Microbiology Reviews*.

[52] Bendy R. H., Nuccio P. A., Wolfe E. (1964). Relationship of quantitative wound bacterial counts to healing of decubiti: effect of topical gentamicin. *Antimicrobial Agents and Chemotherapy (Bethesda)*.

[53] Breidenbach W. C., Trager S. (1995). Quantitative culture technique and infection in complex wounds of the extremities closed with free flaps. *Plastic and Reconstructive Surgery*.

[54] Krizek T. (1967). Bacterial growth and skin graft survival. *Surgical Forum*.

[55] Levine N. S., Lindberg R. B., Mason A. D., Pruitt B. A. (1976). The quantitative swab culture and smear: a quick, simple method for determining the number of viable aerobic bacteria on open wounds. *The Journal of Trauma*.

[56] Robson M. C., Heggers J. P., Klingbeil J. R. (1969). Bacterial quantification of open wounds. *Plastic and Reconstructive Surgery*.

[57] Robson M. C., Heggers J. P. (1970). Delayed wound closures based on bacterial counts. *Journal of Surgical Oncology*.

[58] Robson M. C., Lea C. E., Dalton J. B., Heggers J. P. (1968). Quantitative bacteriology and delayed wound closure. *Surgical Forum*.

[59] Greer L. F., Szalay A. A. (2002). Imaging of light emission from the expression of luciferases in living cells and organisms: a review. *Luminescence: The Journal of Biological and Chemical Luminescence*.

[60] Garcez A. S., Núñez S. C., Azambuja N. (2013). Effects of photodynamic therapy on gram-positive and gram-negative bacterial biofilms by bioluminescence imaging and scanning electron microscopic analysis. *Photomedicine and Laser Surgery*.

[61] Vecchio D., Dai T., Huang L., Fantetti L., Roncucci G., Hamblin M. R. (2013). Antimicrobial photodynamic therapy with RLP068 kills methicillin-resistant Staphylococcus aureus and improves wound healing in a mouse model of infected skin abrasion PDT with RLP068/Cl in infected mouse skin abrasion. *Journal of Biophotonics*.

[62] Andreu N., Zelmer A., Wiles S. (2011). Noninvasive biophotonic imaging for studies of infectious disease. *FEMS Microbiology Reviews*.

[63] Czigany Z., Hata K., Lai W. (2019). A dual protective effect of intestinal remote ischemic conditioning in a rat model of total hepatic Ischemia. *Clinical Medicine*.

